# Social defeat: Vagal reduction and vulnerability to ventricular arrhythmias

**DOI:** 10.1016/j.ynstr.2020.100245

**Published:** 2020-08-03

**Authors:** Charly Brouillard, Pascal Carrive, Caroline Sévoz-Couche

**Affiliations:** a Sorbonne Université, INSERM, UMRS1158 Neurophysiologie Respiratoire Expérimentale et Clinique, F-75005, Paris, France; b Blood Pressure, Brain and Behavior Laboratory, School of Medical Sciences, University of New South Wales, Sydney, NSW, Australia

**Keywords:** Social defeat, Parasympathetic, Brain-derived neurotrophic factor, Stress, Ventricular premature beats

## Abstract

Previously, a sub-population of defeated anesthetized rats (Dlow) was characterized by persistent low blood levels of brain-derived neurotrophic factor (BDNF) at day 29 and autonomic alteration at day 30 after social challenge, while the other population (Dhigh) was similar to non-defeated (ND) animals. The aims of this study were to determine the time-course of autonomic dysfunction in awake animals, and whether Dhigh and/or Dlow were vulnerable to cardiac events. Defeated animals were exposed to four daily episodes of social defeats from day 1 to day 4. At day 30, anesthetized Dlow displayed decreased experimental and spontaneous reflex responses reflecting lower parasympathetic efficiency. In addition, Dlow but not Dhigh were characterized by left ventricular hypertrophy at day 30. Telemetric recordings revealed that Dlow had increased low frequency-to-high frequency ratio (LF/HF) and diastolic (DBP) and systolic (SBP) blood pressure, associated with decreased HF and spontaneous baroreflex responses (BRS) from day 3 to day 29. LF/HF, DBP and SBP recovered at day 5, and HF and BRS recovered at day 15 in Dhigh. Ventricular premature beats (VPBs) occurred in Dlow and Dhigh animals from day 5. Time course of VBP fluctuations in Dhigh mirrored that of HF and BRS, but not that of LF/HF, DBP and SBP.

These results suggest that a psychosocial stress associated to low serum BDNF levels can lead to vulnerability to persistent autonomic dysfunction, cardiac hypertrophy and ventricular ectopic beats. The parasympathetic recovery seen in Dhigh may provide protection against cardiac events in this population.

## Highlights

• Defeated animals are separated based on their levels of BDNF 29 days after the first session of social defeat (day 29).
• Animals with low (Dlow) but not high (Dhigh) BDNF levels present a cardiac hypertrophy and low parasympathetic activity.
• Time course of ventricular premature beats in Dlow and Dhigh follows the reduction in parasympathetic efficiency.
• Parasympathetic recovery in Dhigh may provide protection against cardiac events.

## Graphical abstract

**Figure:**
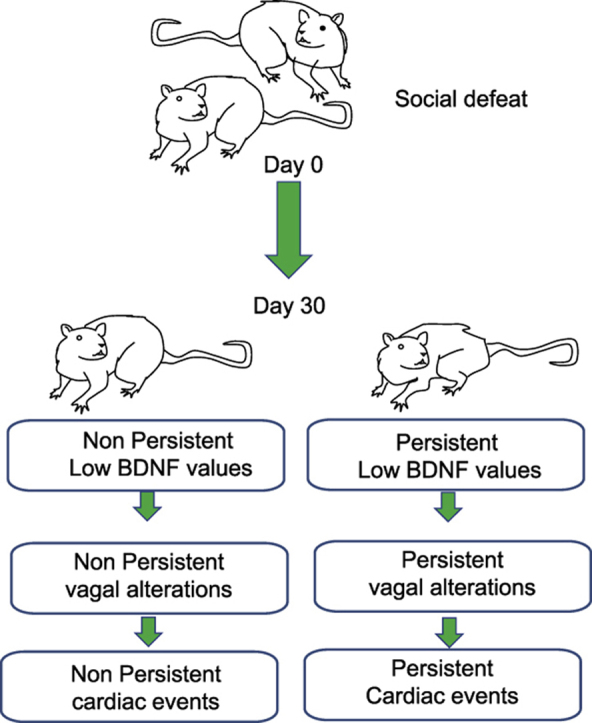

## 1 Introduction

Correlations between psychosocial factors and cardiovascular abnormalities have been the focus of several studies in the past (Rozanski et al., 1999; Sgoifo et al., 1999; Padley et al., 2005). A number of studies have been conducted in animal models. Some of them used experimentally (Moffitt et al., 2002) or genetically (Padley et al., 2005) modified animals, while others used systems more closely resembling the physiological reactions observed in humans following the application of chronic mild stress (Grippo et al., 2004, 2008). Analyses of BRS during or at the end of a chronic mild stress suggested that this parameter was not affected (Grippo et al., 2008; Porter et al., 2004).

Recent advances have shown that social defeat (SD) is one of the most severe known stressors (Koolhaas et al., 1997), and the most appropriate means of evoking sympathetic activation (Costoli et al., 2004; Grippo et al., 2004, 2008) and vagal inhibition (Nalivaiko et al., 2009; Sévoz-Couche et al., 2013). We have previously shown that lower heart rate variability (HRV) and baroreceptor-heart rate (HR) reflex linked to anxiety persisted in defeated animals for at least 6 days after the end of a four-day social challenge (day 10, Sévoz-Couche et al., 2013). More recently, we have identified a sub-population of defeated animals (Dlow), which is characterized by low Brain Derived Neurotrophic Factor (BDNF) serum levels until 25 days after the last of the four session social challenge (day 29), compared to the rest of the population of defeated animals (Dhigh) (Brouillard et al., 2019). Persistent autonomic alterations (lower HRV and spontaneous baroreflex responses) at day 30 were observed in anesthetized Dlow (considered as vulnerable to stress) but not Dhigh (considered as resistant to stress) animals; however, as anesthetics may modify cardiovascular values, alteration of HRV and baroreflex responses in Dlow may be different in awake animals. The first aim of the study was to evaluate continuous HRV and baroreflex responses using telemetric recordings because our hypothesis was that we should observe the same alterations in vigil than in anesthetized animals; this procedure would also estimate the latency of cardiovascular changes in Dlow and Dhigh, and the time of recovery in Dhigh.

Reduction of heart rate variability (HRV) and the baroreceptor-heart rate (HR) reflex were shown to be predictive of ventricular fibrillation occurrence (Billman et al., 1982; La Rovere et al., 2001). Mood disorders are associated with ventricular arrhythmia (Verrier and Lown, 1984; Francis et al., 2009). A solid body of evidence link the autonomic nervous system to life-threatening arrhythmia and death in these diseases (Watkins et al., 1998; Friedman, 2007; Brugada, 2012). SD increased cardiac weight (Morais-Silva et al., 2019) and susceptibility to spontaneous (Sgoifo et al., 1998, 2002; Stilli et al., 2001; Nalivaiko et al., 2009) or experimentally induced ventricular arrhythmias (Grippo et al., 2004) during and a few minutes after stress exposure. Occurrence of cardiac events may be explained by ventricular fibrosis (Costoli et al., 2004) and altered myocardial electrical stability in a potentially proarrhythmic manner (Carnevali et al., 2013). The second aim of the study was to evaluate whether ventricular arrhythmia associated with cardiac hypertrophy may occur and persist in defeated animals after SD challenge, concomitantly with cardiovascular changes. Our hypothesis was that the time-course profile of cardiac events may mirror that of cardiovascular changes in Dlow and Dhigh.

To answer these questions, we performed a four-day social challenge and recorded cardiovascular modifications (HRV and baroreflex alterations) and occurrence of ventricular premature beats (VPBs) using telemetric recordings from before (day −3) to 25 days after the last day (day 29) of SD.

## 2 Materials and methods

### 2.1 General procedures

Experiments were carried out in Sprague Dawley male rats (n = 31), obtained from Centre d’Elevage R. Janvier, Le Genest-Saint-Isle, France) weighing 290–310 g (6–8 weeks old). Animals were housed in individual cages (length x width x height: 45 × 25 × 17 cm) for 1 week before the beginning of the experiments. Wildtype Groningen (WTG) male rats (400–500 g) served as resident rats in confrontation encounters (Sgoifo et al., 1998). These rats were originally bred at the University of Groningen (The Netherlands) under conventionally clean conditions. The same WTG rats were used for all successive series of defeat episodes. All animals were kept under controlled environmental conditions (i.e. 22 ± 1 °C, 60% relative humidity, 12-h light [7:00–19:00] and 12-h dark [19:00–7:00] cycles, ad libitum food and water). Procedures involving animals and their care were all performed in accordance with the institutional guidelines, which follow national and European laws and policies (2010/63/EU). Ethical approval was obtained for this project (#2493).

#### 2.1.1 Social defeat paradigm

**Fig. 1:**
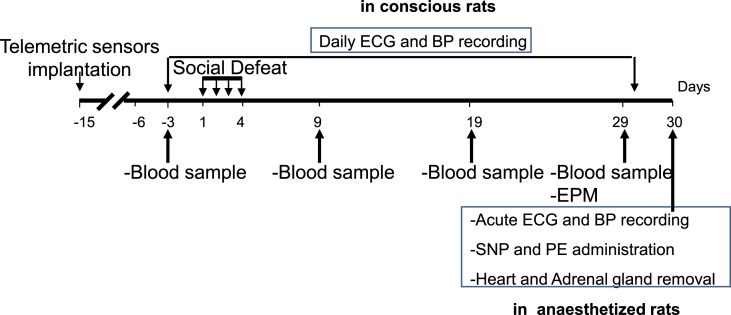
**Experimental timeline of the experiment.** EPM was performed at Day 9 and day 29, and HRV parameters, HR and MAP were obtained under anesthesia at Day 30. Blood samples were taken at day −3 and day 29 to compare BDNF values at these two time points.

SD consisted of four conditioning sessions (one per day, day 1 to day 4, Fig. 1) that involved the same pairs of residents and intruders (Becker et al., 2001; Claverie et al., 2016). The 45 min sessions were divided into two consecutive periods. During the first period (30 min), intruders were placed individually in a protective cage within the resident animal's home cage. The protective cage allowed unrestricted visual, auditory and olfactory contact with the resident but prevented close physical contact. During the second period (15 min), the protective cage was removed, either with the resident remaining present, which allowed for physical confrontation with the intruder (3–4 confrontations of ~10 s, during each of which the intruding (defeated) animal was always dominated by the resident rat) or with the resident removed, which gave the intruder access to the entire resident home cage (non-defeated control intruders). The control intruders were therefore never physically attacked and defeated by the resident and didn't show any sign of stress and/or anxiety. Animals that were wounded as a result of the SD procedure were excluded from the experiment (2% of stressed animals)

#### 2.1.2 Elevated plus maze (EPM) test

We used an EPM test (described in details elsewhere, Rivat et al., 2010; Brouillard et al., 2019) to evaluate anxiety-related behaviour in animals (Lee and Rodgers, 1990) at day 9 and day 29. Briefly, the rats' behavior was videotaped with an LCD camera connected to control and recording equipment located in the adjacent room. The time spent in the various arms (open, closed or center) and the numbers of entries into the open and closed arms of the plus-maze were recorded with custom-made software, as indicators or anxiety state. Total number (open + closed, Rivat et al., 2010) of arm entries was also calculated. EPM was performed between 9am and 11am, so animals were allowed 2h to recover before telemetric recordings (see below). No effects on cardiac values should be evoked by EPM because this procedure doesn't affect HR stability by itself (Henze et al., 2008)

#### 2.1.3 Adrenal gland and heart weight evaluation

At the end of experiments (day 30), adrenal glands and heart were removed and weighed. Organ weights were expressed relative to total body weight (in mg 100 g^−1^ and mg g^−1^ body weight, respectively)

#### 2.1.4 BDNF analysis and K means clustering

Blood samples (200 μl) were collected from the tail vein at different time points (day −3 and day 29) in awake rats (Claverie et al., 2016). After centrifugation, the serum was separated and stored at −20 °C. Serum BDNF concentrations were determined using a 1:25 dilution in blood and a 1:10 dilution in the RVLM using a commercial assay (Promega Corporation, Madison, WI, USA) in 96-well plates (Corning Costar® EIA plate, New York, NY, USA) in accordance with the manufacturer's instructions. The BDNF concentration was expressed in ng ml^−1^

Separation of defeated animals in Dlow and Dhigh based on their BDNF levels at day −3 and day 29 was performed using the K-means algorithm method (RealStats, Excel).

After arbitrary assignment of each animal to one of two Clusters and calculation of K1 and K2 centroids (average of all attribute values in a specific Cluster, to minimizing the square of the distance) for each Cluster (Asghar et al., 2019; Bystrzanowska et al., 2002), squared Euclidean Distances (D, “ordinary” straight-line distance between two points), were obtained as follow:

D1^2^=(X–K1_X_)^2^ + (Y–K1_Y_)^2^

D2^2^=(X–K2_X_)^2^ + (Y–K2_Y_)^2^

with D1: squared Euclidian Distance from centroid K1, D2: squared Euclidian Distance from centroid K2, X: BDNF at day −3, and Y: BDNF at day 29.

Animals were reassigned to the specific Cluster with the shortest distance (i.e. if D1 was smallest than D2, then the animal was assigned to Cluster 1) and new centroids based on the average of all new attribute values in the specific Cluster were calculated.

Stopping criterion was a maximum of 10 iteration or below if reassignment and centroids were similar to those obtained in the preceding iteration.

### 2.2 General procedures for the measurement of cardiovascular parameters in anesthetized animals

#### 2.2.1 Femoral cannulations and ECG recordings

At day 30 (Fig. 1A), the rats were anesthetized with pentobarbital sodium (Ceva Santé Animale, Libourne, France; 60 mg/kg i.p.) (Brouillard et al., 2019). Systemic mean blood pressure (MBP) was monitored via a femoral artery catheter. Electrocardiogram (ECG) was recorded using stainless steel pins placed subcutaneously into the forepaws and hind paws. These signals were amplified, filtered and relayed to a computer running Spike 2 (version 6.08) software (CED). The depth of anesthesia was assessed regularly by pinching a hind paw and monitoring the stability of MBP and HR recordings.

#### 2.2.2 Pharmacological baroreflex activation and evaluation

The bolus administration of sodium nitroprusside (SNP, 100 g kg^−1^, i.v.) followed by phenylephrine (PE, 10 g kg^−1^, i.v.) made it possible to generate baroreceptor function curves, by fitting a sigmoid logistic function to the data (Netzer et al., 2011; Sévoz-Couche et al., 2013). Cardiac baroreceptor reflex responses were evaluated at the maximum (peak changes) responses. Maximal baroreflex changes in HR (BRR) were quantified and plotted as changes in heart rate over changes in mean arterial pressure (maximal BRR: ΔHR/ΔMBP, beats per minute mmHg^−1^). The standard and rectilinear baroreflex slopes were also calculated from the baroreceptor curves (Netzer et al., 2011).

#### 2.2.3 Spontaneous baroreflex evaluation

We used the sequence method to calculate spontaneous baroreflex response (BRS) to spontaneous blood pressure changes (Laude et al., 2004; Sévoz-Couche et al., 2013; Brouillard et al., 2019). BRS was calculated as the mean slope of R—R interval sequences for all sequences detected during 90 s segments of data

#### 2.2.4 HRV analysis

Power spectra of HRV were calculated using fast Fourier transformation (size 256, Hanning window (Padley et al., 2005)), with a sample interval of 0.2 s to obtain a power spectral density over 2.5 Hz. HRV was performed on the time interval between two consecutive beats (RR interval) derived from ECG, for each 4-min time period of telemetry recording (see below), using Spike 2 (version 6.08) software (CED). Low frequency (LF) and high frequency (HF) powers (absolute values) were determined within the 0.2–0.7 and 0.7–2.5 Hz frequency ranges, respectively (Sévoz-Couche et al., 2013). LF-to-HF ratio (LF/HF), normalized LF (LFnu: LF/[LF + HF]*100) and HF (HFnu: HF/[LF + HF]*100) were also calculated. LF and HF domains are under the influences of both branches of the autonomic nervous systems (Berntson et al., 1997; Pagani et al., 1997). HF power (that includes the respiratory sinus arrhythmia) is known to be exclusively under parasympathetic (vagal) control while the LF band is a reflection of both sympathetic and parasympathetic influences on HRV. HFnu and LFnu are believed to represent parasympathetic and sympathetic influences, respectively

### 2.3 General procedures for the measurement of cardiovascular parameters in awake animals

Two weeks before SD (day −15, Fig. 1), rats were implanted with radio-telemetric probes (Data Sciences International, St. Paul, MN) to enable recording of ECG, MBP, diastolic blood pressure (DBP) and systolic blood pressure (SBP) (Brouillard et al., 2016). Briefly, the probes were implanted in the peritoneal cavity, and ECG wires were tunnelled subcutaneously along the rib cage. Canula for blood pressure recordings was placed in the aorta. Dataquest A.R.T 4.1 software was used to record telemetry signals every afternoon (1–6 p.m.) during the week from day −3 to day 29. The afternoon was chosen for two main reasons. First, recordings are more stable when animals are less active. Second, SD procedure took place early in the morning, so telemetric recordings started at least 2 h after the stress sessions.

Signals were transmitted for 4 min every 2 min during each data collection period. Waveform data were imported off-line into Spike 2 (version 6.08) software (CED) BRS, HR and HRV were determined as mentioned above. In addition, the number of the most common ventricular arrhythmic events, i.e. VPBs, was analysed (Sgoifo et al., 1998). After visual inspection of ECG recordings to remove artifacts, VPBs was determined and quantiﬁed ofﬂine. According to the Lambeth Conventions for the study of experimental arrhythmias (Curtis et al., 2013). VPBs were first identified by using a script from CED based on QRS enlargement. Then artifacts were removed and VPBs was counted when associated with absence of P wave, longer time (compensatory pause) after ventricular beat wave and concomitant blood pressure decrease.

### 2.4 Statistical analysis

A K-means clustering method based on serum BDNF values obtained before social defeat (Day −3) and at the end of the procedure (Day 29) was first used to subdivide the defeated animals into those with lower (Dlow) and higher (Dhigh) BDNF level subgroups at D30 (Claverie et al., 2016; Brouillard et al., 2019). The three groups (ND, Dlow and Dhigh) were then compared using ANOVAs. Differences between BDNF values at day −3 and day 29 were evaluated using a Paired Student's *t*-test

Differences between the three groups at day 30 in heart and gland weight, as well as HR, MBP, BRS, maximal BRR, and power spectral parameters were calculated in anesthetized animals, using a one-way ANOVA. Baroreflex linear slopes were analysed with unpaired *t*-test.

Differences between the three groups in BDNF at day −3 and day 29, and in EPM at day 9 and day 29, and longitudinal changes in MBP, DBP, SBP, HR, LF/HF, LF, LFnu, HF, HFnu, BRS and arrhythmia (day −3 to day 29), were assessed with repeated measure two-way ANOVA where the repeated factor was time and the other factor was defeat (ND, Dlow, Dhigh).

Post hoc analysis with Bonferroni's multiple comparisons test was applied after ANOVA when necessary, and results were considered significant if *P* < 0.05. Analyses were performed using Prism 8.11 (GraphPad Software).

## 3 Results

### 3.1 Identification of subgroups of defeated animals based on the K-means clustering between day −3 and day 29

**Fig. 2:**
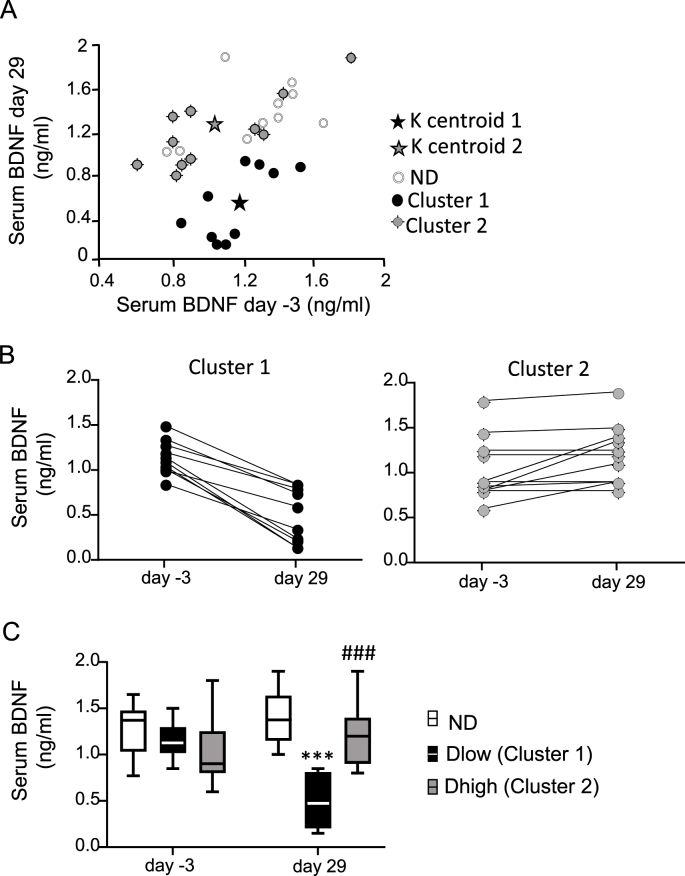
**Classification of defeated animals in Dlow or Dhigh subgroups**. A. K-means clustering method based on baseline (day −3) and recovery (day 29) serum BDNF levels. Defeated animals were separated in Cluster 1 and Cluster 2. Open circles: Non-defeated (ND) animals. B. Individual values of defeated animals in Cluster 1 and Cluster 2 showing that compared to day −3, BDNF values were lower at day 29 in Cluster 1 (animals were identified as Dlow) but not in Cluster 2 (animals were identified as Dhigh). C. BDNF values were not different between ND, Dlow and Dhigh at day −3, but Dlow had lower values than Dhigh and ND at day 29. Box and whisker graphs indicate the minimum and maximum values and the median. ***p < 0.001 versus ND, ^###^p < 0.001 versus Dlow, Bonferroni's post hoc analysis.

With the use of the K-means clustering method (Claverie et al., 2016; Brouillard et al., 2019), we identified two Clusters of defeated animals (n = 21) when the algorithm stopped after 6 iterations. Coordinates were (X:1.17; 0.55) for final centroid of Cluster 1 and (X: 1.05; Y: 1.31) for final centroid of Cluster 2 (Fig. 2A). Taking into account previous results (Claverie et al., 2016; Brouillard et al., 2019), and because individual values of BDNF were lower at day 29 than at day −3 (mean value: 0.49 ± 0.09 vs 1.15 ± 0.06, respectively, P < 0.0001), animals (n = 10) in Cluster 1 were identified as the Dlow group (Fig. 2B). All defeated animals in Cluster 2 (n = 11) had similar or increased BDNF values at day 29 compared to day −3 (mean value: 1.20 ± 0.09 vs 1.03 ± 0.10, respectively, P = 0.02), and were considered Dhigh (Fig. 2B).

A group effect and interaction between time and groups was seen for BDNF values (Table 1). Dlow values were not different to ND and Dhigh at day −3 but were lower to both groups at day 29 (Fig. 2C). Dhigh and ND had similar values at day −3 and day 29.

**Table 1:** Two-Way ANOVA Statistics for physiologic and autonomic parameters.

Parameters	Group effect	Time effect	Interaction
BDNF	F (2, 28) = 8.8 P = 0.0010	F (1, 28) = 7.2 P = 0.0118	F (2,28) = 37.2 P < 0.0001
EPM closed	F (2,28) = 14.9 P < 0.0001	F (1,28) = 51.4 P < 0.0001	F (2,28) = 3.6 P = 0.04
EPM open	F (2,28) = 40.9 P < 0.0001	F (1,28) = 25.4 P < 0.0001	F (2,28) = 3.5 P = 0.04
EPM center	F (2,28) = 2.9 P = 0.06	F (1,28) = 25.4 P < 0.0001	F (2,28) = 0.41 P = 0.66
EPM	F (2,28) = 7.2	F (1,28) = 51.6	F (2,28) = 21.92
Total entries	P < 0.0001	P < 0.0001	P < 0.0001
HR	F (2, 28) = 1.526; P = 0.235	F (10, 280) = 50.87; P < 0.001	F (20, 280) = 1.465; P = 0.090
DBP	F (2, 28) = 9.619; P = 0.0007	F (10, 280) = 2.527; P = 0.0063	F (20, 280) = 2.126; P = 0.003
SBP	F (2,28) = 5.360; P = 0.0107	F (10, 280) = 2.583; P = 0.0052	F (20, 280) = 2.170; P = 0.003
MBP	F (2, 28) = 6.776; P = 0.0040	F (10, 280) = 3.366; P = 0.0004	F (20, 280) = 2.535; P = 0.004
LF/HF	F (2, 28) = 13.54; P = 0.00010	F (10, 280) = 3.767; P=<0.0001	F (20, 280) = 2.629; P = 0.004
LF	F (2, 28) = 0.121; P = 0.885	F (10, 280) = 1.203; P = 0.288	F (20, 280) = 1.209; P = 0.245
LFnu	F (2, 28) = 13.74; P < 0.0001	F (10, 280) = 3.646; P = 0.0001	F (20, 280) = 1.787; P = 0.04
HF	F (2, 28) = 11.74; P = 0.0002	F (10, 280) = 2.837; P = 0.0022	F (20, 280) = 3.619; <0.0001
HFnu	F (2, 28) = 11.59; P = 0.0002	F (10, 280) = 3.468; P = 0.0003	F (20, 280) = 1.616; P = 0.04
BRS	F (2, 28) = 21.31; P < 0.0001	F (10, 280) = 7.100; P < 0.0001	F (20, 280) = 2.905; P < 0.0001
VPB	F (2, 28) = 30.17; P < 0.0001	F (10, 280) = 5.613; P < 0.0001	F (20, 280) = 2.633; P = 0.0002

Two-way repeated measures ANOVA statistics for BDNF levels, time spent in open, closed and center arms in Elevated Plus-Maze (EPM) test, total entries (closed + open arms) in EPM, frequential HRV (LF/HF, LF, HF, normalized LF [LFnu] and normalized HF [HFnu]), mean blood pressure (MBP), diastolic blood pressure (DBP), systolic blood pressure (SBP), heart rate (HR), baroreflex spontaneous response (BRS), and ventricular premature beats (VPB).

### 3.2 Behavioural and physiological changes at day 9, day 29 and day 30

No interaction was found for time spent in EPM center arms (Table 1).

A group effect and interaction between time and groups was seen for time spent and total number of entries in open and closed arms in EPM (Table 1). At day 9, the amount of time spent by Dlow and Dhigh was larger in closed arms (Supplemental Fig. 1A) and smaller in open arms (Supplemental Fig. 1B) compared to ND. Time spent in both arms were similar in Dlow and Dhigh. Total number of entries was lower in Dlow compared to ND and Dhigh, and was similar between Dhigh and ND (Supplemental Fig. 1D). At day 29, there was no difference between groups in amount of time spent or in total number of entries in EPM arms (Supplemental Figs. 1A–D).

At day 30, ND, Dlow and Dhigh had similar adrenal gland weight [F (2, 28) = 1.55; P = 0.2263] (Supplemental Fig. 1E).

**Fig. 3:**
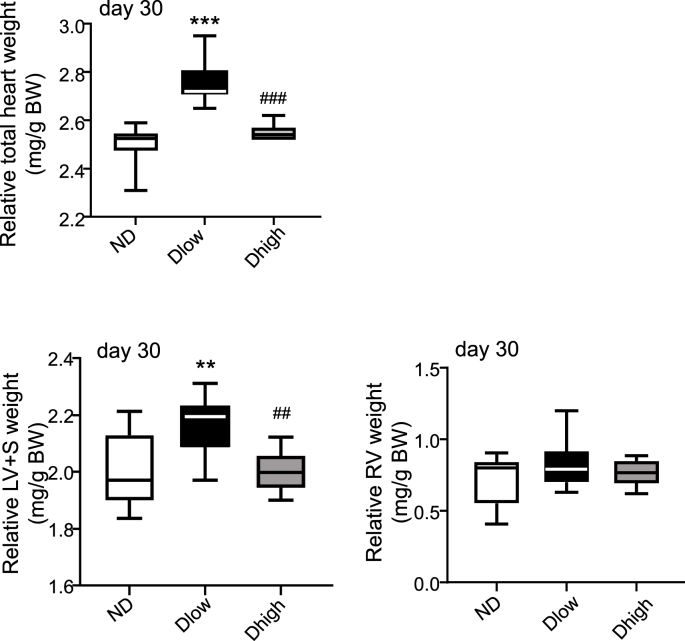
**Dlow present a left ventricular hypertrophy at day 30. Evaluation of cardiac hypertrophy**. Relative total heart, left ventricle plus septum (LV + S) and right ventricle (RV) weights showed that Dlow presented a LV hypertrophy at the end of the procedure. Box and whisker graphs indicate the minimum and maximum values and the median. ***p < 0.001 versus ND. ^###^p < 0.001 versus Dlow.

At day 30, the relative total heart [F (2, 28) = 53.15; P < 0.0001] and left ventricle plus septum (LV + S) [F (2, 28) = 7.70; P = 0.0021] weights were increased in Dlow compared to ND and Dhigh (Fig. 3). The relative right ventricle (RV) weight was not different in the three groups [F (2, 28) = 1.34; P = 0.27] (Fig. 3).

### 3.3 Baroreflex responses and HRV in anesthetized animals at day 30

**Fig. 4:**
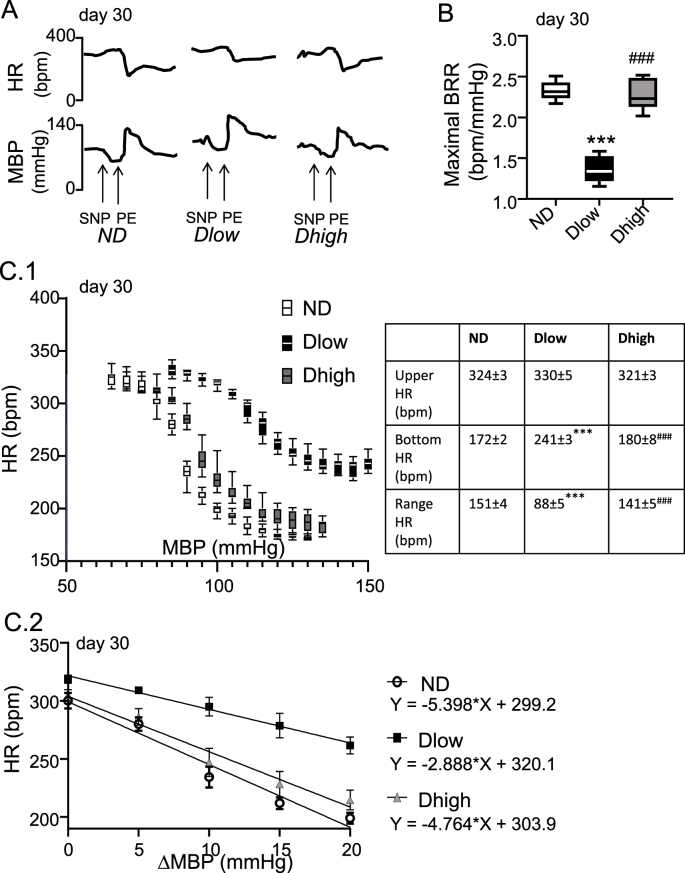
**Dlow have a reduced cardiac response to experimentally-induced baroreflex**. A. Representative traces showing the global cardiac modification induced by sodium nitroprusside (SNP) followed by phenylephrine (PE). B. The maximal baroreflex cardiac response (BRR: ΔHR/ΔMBP) was reduced in Dlow compared to ND and Dhigh rats. Box and whisker graphs indicate the minimum and maximum values and the median. ***p < 0.001 versus ND. ^###^p < 0.001 versus Dlow. C. C1: Curves of cardiac baroreceptor gain. Reﬂex fall in HR (y-axis) in response to increase in MBP (x-axis) triggered by SNP + PE administration is lower in Dlow than in Dhigh and ND. Parameters of the baroreceptor curves for ND, Dlow and Dhigh rats are given in Table. C2: Slopes were calculated by regression analysis of the rectilinear part of the baroreceptor curves. Slope in Dlow is lower than in Dhigh and ND.

We induced experimental pharmacological baroreflex through bolus administration of SNP followed by PE at day 30 (Fig. 4A). Resting HR was not different between groups [F (2, 28) = 1.23; P = 0.3075] but MBP was higher in Dlow [F (2, 28) = 14.12; P < 0.0001] (Supplemental Figs. 2A–B). Maximal reflex cardiac changes due to pressure modifications following SNP plus PE were reduced [F (2, 28) = 133.1; P < 0.0001] in Dlow compared to ND and Dhigh (Fig. 4B). Sigmoid logistic function added to the data showed that Bottom HR [F (2, 28) = 396.5; P < 0.0001] and Range HR [F (2, 28) = 123.2; P < 0.0001], but not Upper HR [F (2, 28) = 3.790; P = 0.4162], were reduced in Dlow compared to other groups (Fig. 4C1). The slope of the linear part of the sigmoid curves was also diminished in Dlow vs ND (P < 0.0074) and Dhigh (P < 0.0112) (Fig. 3C2). Compared to ND and/or Dhigh, Dlow had lower BRS [F (2, 28) = 55.24; P < 0.0001, Supplemental Fig. 2C]. In addition, LF/HF ratio [F (2, 28) = 17.85; P < 0.0001] and LFnu [F (2, 28) = 16.52; P < 0.0001] but not LF [F(2, 28) = 0.56, P = 0.57] were higher in Dlow than ND and Dhigh; HF [F (2, 28) = 3.439; P = 0.0462] was lower in Dlow than Dhigh but not ND, and Dlow HFnu (F (2, 28) = 17.01; P < 0.0001) was lower than both ND and Dhigh, (Supplemental Fig. 3). Dhigh and ND were similar for all values (Supplemental Fig. 3).

### 3.4 Time course changes in autonomic parameters and ventricular arrhythmias

Daily telemetric ECG recording were performed across day −3 to day 29.

**Fig. 5:**
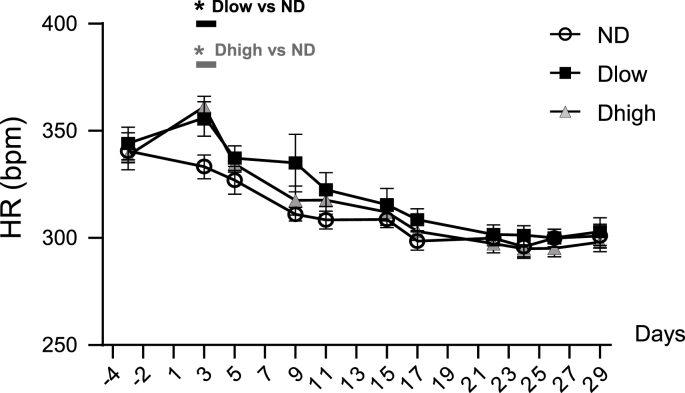
**Time-course profile of heart rate show no long-lasting increases in Dlow or Dhigh**. Changes in heart rate (HR) before, during and after social defeat up until day 29. HR was higher in Dlow and Dhigh than in ND at day 3 but recovered as early as day 5. Values are the mean ± SEM. Symbols over horizontal bars indicate when groups were significantly different (p < 0.05, Bonferroni's post hoc analysis).

There was no group effect or interaction effect for HR (Fig. 5 and Table 1).

**Fig. 6:**
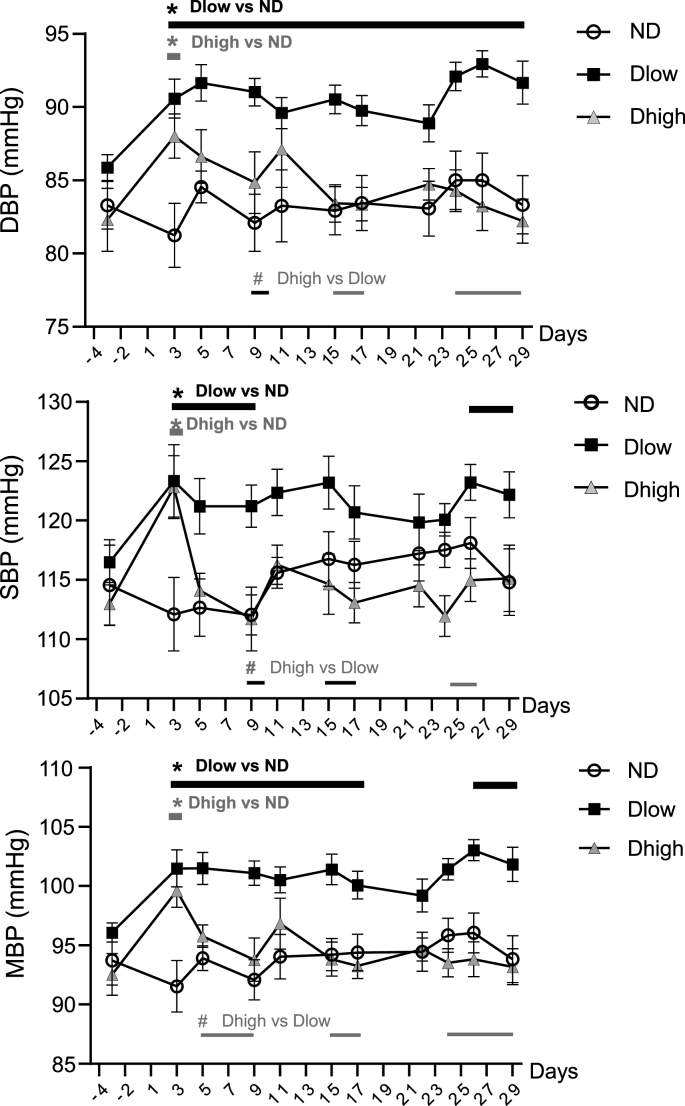
**Time-course profile of blood pressure show long-lasting increases in Dlow compared to Dhigh**. Changes in diastolic (DBP), systolic (SBP) and mean (MBP) blood pressure before, during and after social defeat up until day 29. These three parameters were higher in Dlow and Dhigh animals than in ND at day 3. Changes in Dlow persisted sporadically until day 29, but lasted until day 5 in Dhigh. Values are the mean ± SEM. Symbols over horizontal bars indicate when groups were significantly different (p < 0.05, Bonferroni's post hoc analysis).

A group effect and interaction between time and groups was seen for DBP, SBP, MBP, BRS and ventricular arrhythmias (Table 1). Compared to ND, Dlow had higher DBP from day 3 to day 29 and higher SBP from day 3 to day 9, and day 26 to day 29 (Fig. 6). Consequently, Dlow MBP increased compared to ND MBP from day 3 to day 29, with a brief interruption from day 19 to day 25. Dhigh had higher DBP, SBP and MBP at day 3 only. Dhigh and Dlow MBP, DBP and SBP were significantly different with some interruptions from day 5 to the end of the experiment.

**Fig. 7:**
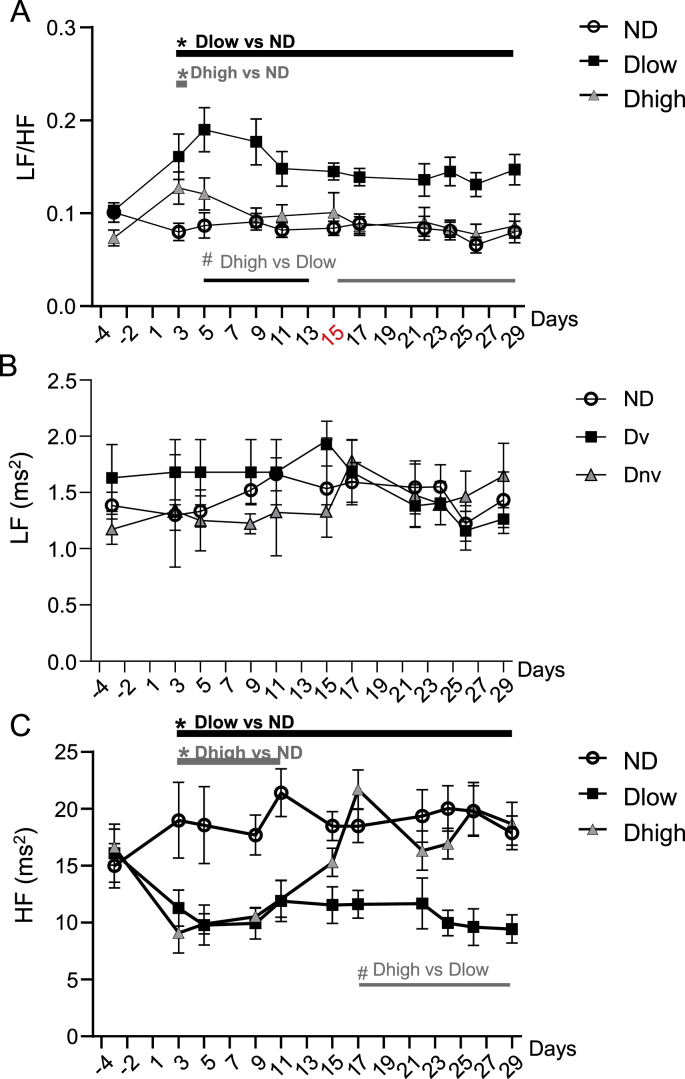
**Time-course profile of frequential HRV show long-lasting alterations in Dlow compared to Dhigh**. Changes in LF/HF, LF and HF before, during and after social defeat up until day 29. LF/HF was higher and HF was lower in Dlow and Dhigh than in ND at day 3. Changes in Dlow persisted until day 29, but LF/HF increase lasted only 1 day and HF reduction was observed only until day 11 in Dhigh. LF didn't change throughout the experiment. Values are the mean ± SEM. Symbols over horizontal bars indicate when groups were significantly different (p < 0.05, Bonferroni's post hoc analysis).

**Fig. 8:**
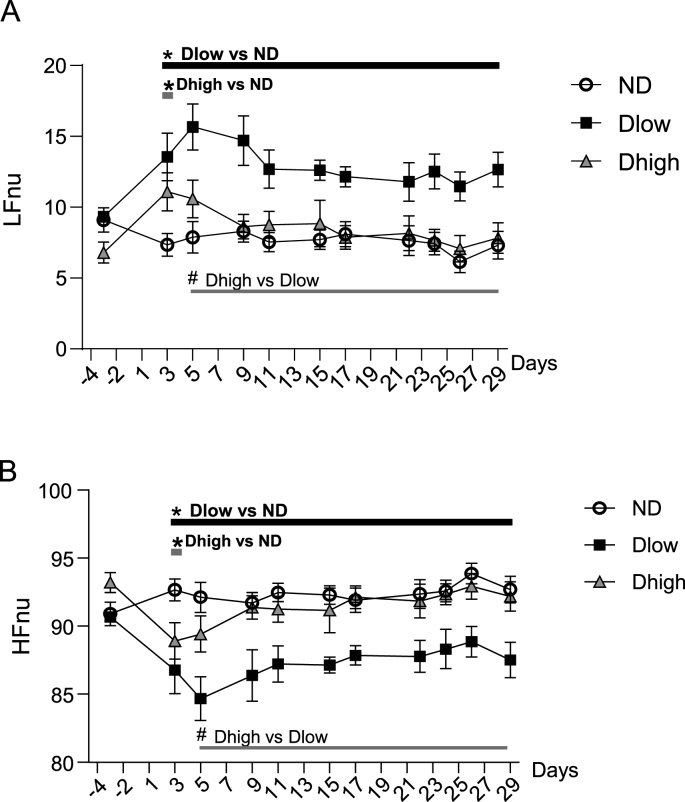
**Time-course profile of normalized LF and HF show long-lasting alterations in Dlow compared to Dhigh**. Changes in normalized LF and HF (expressed in normalized units) before, during and after social defeat up until day 29. LFnu was higher and HFnu was lower in Dlow and Dhigh than in ND at day 3. Changes in Dlow persisted until day 29, but those in Dhigh recovered by day 5. Values are the mean ± SEM. Symbols over horizontal bars indicate when groups were significantly different (p < 0.05, Bonferroni's post hoc analysis).

A group effect and interaction between time and groups was seen for LF/HF, HF, LFnu and HFnu, but not LF (Table 1). Compared to ND, Dlow had higher LF/HF from day 3 to day 29 (Fig. 7A). Dhigh ratio was higher than ND ratio at day 3 only. Dhigh and Dlow ratio were statistically different from day 5 to day 29. Compared to ND, Dlow and Dhigh had similar LF absolute values (Fig. 7B), but Dlow had lower HF from day 3 to day 29 (Fig. 7C). Dhigh HF was reduced compared to ND HF from day 3 to day 11. Dhigh and Dlow HF were significantly different from day 17 to day 29 (Fig. 7C). Compared to ND, Dlow had higher LFnu and lower HFnu from day 3 to day 29 (Fig. 8A and B). LFnu was higher and HFnu was lower in Dhigh than ND at day 3 only, and these values were different from Dlow from day 5 to day 29.

**Fig. 9:**
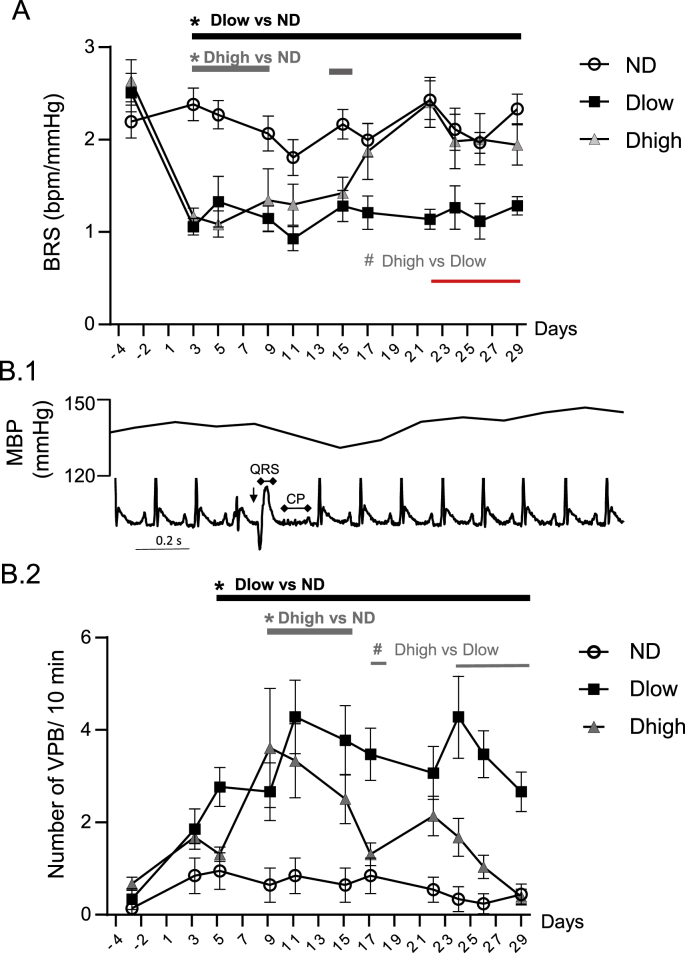
**Time-course profile of baroreflex and ventricular arrhythmias show similar long-lasting fluctuations in Dlow compared to Dhigh**. A. Spontaneous baroreflex response (BRS) was lower in Dlow and Dhigh animals than in ND at day 3. BRS reduction persisted in Dlow until day 29, but lasted only until day 9 in Dhigh. B1. Ventricular premature beats (VPBs) were identified with enlargement of the QRS complex, increase in the compensatory pause (CP) and the absence of P wave (arrow). MBP decreased concomitantly to the occurrence of VPBs. B2. The number of VPBs was higher in Dlow at day 5 and in Dhigh at day 9 than in ND. VPBs increases persisted in Dlow until day 29, but lasted only until day 15 in Dhigh. Values are the mean ± SEM. Symbols over horizontal bars indicate when groups were significantly different (p < 0.05, Bonferroni's post hoc analysis).

Compared to ND, Dlow had lower BRS from day 3 to day 29 (Fig. 9A). Dhigh BRS was lower than ND BRS from day 3 to day 9, and recovered at day 11. Dhigh and Dlow BRS were different from day 17 to day 29.

Occurrence of ventricular premature beats (VPBs, Fig. 9B1) mirrored time course of reduction in HF and BRS (Fig. 9B2). Compared to ND, Dlow had higher number of VPBs from day 5 to day 29. Dhigh VPBs increased compared to ND VPBs from day 9 to day 15. Dhigh and Dlow VPBs were different from day 17 to day 29, apart for a brief interruption between day 19 and day 23.

## 4 Discussion

Here we present a full description of autonomic alterations in defeated animal vulnerable to arrhythmias (Dlow). These animals were characterized by permanent low BDNF levels and presented not only with vagal reduction (modified indices of frequential HRV, spontaneous and pharmacological baroreflex responses), but also with cardiac hypertrophy, compared to the other defeated animals (Dhigh). Importantly, the time course of cardiovascular changes suggests that vagal reduction coincided with occurrence and maintenance of ventricular cardiac events.

Animal models of depression and psychosocial stress, including social defeat, are associated with increased BDNF levels in the medulla or the forebrain (Chan et al., 2010; Clark et al., 2011; Greenberg et al., 2014). On the other hand, reduced hippocampal neurogenesis, hippocampal dendritic arborization, and hippocampal BDNF were also observed in these models (Castren et al., 2007; Alleva and Francia, 2009). Treatment with Dammarane Sapogenins improved BDNF expression in the hippocampus and prefrontal cortex, and reduced anxiety-like and depression-like behaviour in mice (Jiang et al., 2019). This is consistent with mood disorder phenotype described in humans (Duman and Monteggia, 2006). A correlation between serum and hippocampal BDNF level reduction in men was previously determined (Kreinin et al., 2015), though the mechanism involved in this association remains unclear. In the short term, low BDNF levels appear to be compensatory, protecting against deleterious chronic stress-related events, rather than maladaptive (Ortiz et al., 2014). Regardless, based on the works from Dr Benoliel's group, there is a clear-cut association between lower levels of hippocampal and circulating BDNF and vulnerability to depression after SD due to local oxidative stress (Blugeot et al., 2011; Bouvier et al., 2017). Social challenge that induced anxiety in defeated rats was associated with autonomic dysfunction, nine days (day 9) after the first of four sessions of social defeat (Sévoz-Couche et al., 2013). Subsequently, using the K-mean method based on circulating BNDF levels (Claverie et al., 2016; Brouillard et al., 2019), we found two Clusters among all defeated animals. Individual values showed that all defeated animals in one Cluster had lower values of BDNF at day 29 than before SD (day −3). This group was called Dlow. Animals from the other Cluster had BDNF values similar at day 29 than at day −3, and were called Dhigh. To the best of our knowledge, it is the first time that individual differences among defeated animals with susceptibility to cardiac abnormalities are based on endogenous parameters (BDNF levels, identification of groups through K-mean clusters) vs phenotype (i.e. depression-like behaviour, Silva-Morais et al., 2019 or dominate/subordinate behaviour, Bartolomucci et al., 2003).

Dlow and Dhigh both developed anxiety at day 9, spending similar lower time in open arms of the EPM, compared to ND. Interestingly, the total number of entries was lower in Dlow but not in Dhigh compared to ND. The number of entries is often described as a reflection of general activity, though a reduced number of entries may be associated with either higher or lower distance travelled in each arm. As only Dlow presented anxiety associated with reduced number of entries in the EPM at day 9, this criterion may represent an early identification of groups. At day 29, the time spent and total entries in EPM was not different between groups, suggesting that no defeated animals exhibited any behaviour modification at that point, though learning and habituation of the situation after a similar test performed at day 9 can't be totally excluded.

Adrenal gland weight at day 30 was similar in ND, Dlow and Dhigh. However, we observed at day 30 a cardiac hypertrophy in Dlow. It was due to increased weight of LV, while RV was not affected. After 15 consecutive days of social challenge, a six-fold larger volume fraction of fibrosis than in control animals was observed in mice (Costoli et al., 2004). In rats, Carnevali et al. (2013) have reported that, despite a longer exposition to stress (12 sessions) than in our procedure (4 sessions), defeated animals presented a modest fibrosis in LV at the end of a social defeat paradigm that lasted 31 days. The fact that no discrimination between potential Dlow and Dhigh groups within defeated rats may have mitigated this finding. Initially, cardiac hypertrophy appears to be an adaptive response to maintain cardiac function after stress and this can explain the hypertrophy found just after a stress procedure (Morais-Silva et al., 2019); however, as time progresses, these changes become maladaptive and the heart may ultimately fail (Tham et al., 2015). Our data indicate that animals with persistent lower BDNF levels were vulnerable to cardiac hypertrophy, and may suggest that Dlow can present heart failure sensitivity. Further studies will be necessary to investigate this possibility.

In a previous study with anesthetized Dlow and Dhigh (Brouillard et al., 2019), HR at day 30 was not different between groups; nevertheless, MBP increased in Dlow compared to the other groups, and autonomic balance was modified in favour of a sympathetic hyperactivity, mainly due to reduced vagal efficiency (low HFnu and BRS). We confirmed the changes in MBP, HFnu and BRS in anesthetized rats in the present study, and extended baroreflex estimation by the analysis of experimentally induced baroreflex. Rats underwent baroreflex unloading and reloading using intravenous bolus SNP followed by PE (Sévoz-Couche et al., 2013). The maximal bradycardia was lower in Dlow, as indicated by a reduction of the HR range. This was the consequence of the bradycardia induced by PE (baroreflex vagal parasympathetic activation, known to be abolished by atropine, Ismay et al., 1979; Comet et al., 2007) because the Bottom HR was less than in other groups. On the contrary, the Upper HR was similar between groups, showing that the first part of the curve, i.e. the tachycardia following vasodilation induced by SNP (Grippo et al., 2008) was not affected. Our results show that social defeat induced in Dlow a reduction of the baroreflex vagal bradycardia, but had no effect on sympathetic baroreflex unloading.

The time course of cardiovascular changes was evaluated in awake animals through telemetric recordings performed each afternoon. HR was slightly increased in Dlow and Dhigh at day 3 but it recovered rapidly in both groups, which is why HR was found unaffected at day 30 in all defeated anesthetized animals. However, MBP increased in Dlow and Dhigh even before the end of the four-day stress procedure (i.e. at day 3). These modifications persisted in Dlow until the end of the protocol, but Dhigh recovered as soon as the stress procedure was over (day 5). We noted that increases in DBP but also SBP, though at a lesser extent, were observed in Dlow. Increase in MBP may be the reflection of sympathetic nerve discharge hyperactivity; however DBP increases linked to mood disorder has been shown to be the consequence of cardiac vagal tone reduction (Yang et al., 2020), so we can't exclude that blood pressure modifications seen after SD, due to a large extent to DBP increase, may be the consequence of parasympathetic reduction more than sympathetic hyperactivity. DBP has traditionally been considered the most important component of blood pressure. DBP increases with age up to 55 years and decreases after that, whereas SBP progressively increases with age at least up to 80 years of age in Western populations (Burt et al., 1995). Consequently, elevated diastolic pressure is more common among young and middle-aging than elderly patients, which is why it is the primary target of antihypertensive therapy. However, over 30 years ago important epidemiological studies pointed out the importance of systolic blood pressure, and research during the 1990s has strengthened this view. Domanski et al. (2002) demonstrated the need to combine systolic and diastolic blood pressure in risk assessment. They concluded that both systolic and diastolic blood pressure increases were better predictors of cardiovascular mortality than change in pulse pressure (due to isolated systolic or diastolic pressure increase).

BRS reduction was linked to vagal dysfunction in anxiety (Watkins et al., 1998). Here we found that BRS changes were concomitant to HF evolution: a reduction was seen from day 3 to day 29 in Dlow, but only from day 3 to day 17 in Dhigh. These results, associated with lower BRR at day 30, confirm a persistent vagal reduction in Dlow while parasympathetic alteration recovered shortly after SD in Dhigh. Analyses of baroreflex responses and frequential HRV showed that reductions in vagally mediated BRS, HF and HFnu (Berntson et al., 1997; Laude et al., 2004) were seen in Dlow and Dhigh at day 3, confirming vagal reduction in all defeated animals at early stages. This alteration persisted until day 29 in Dlow, but Dhigh recovered to initial values around day 15. The LF/HF ratio had the same time course as DBP. Dlow presented a persistent and uninterrupted increase from day 3 to day 29, but Dhigh recovered as early as day 5. These results confirm that Dhigh presented shorter-lived modifications than Dlow. LF (influenced by both sympathetic and parasympathetic activities) was not modified in Dlow compared to both ND and Dlow so the increase in LF/HF was mainly due to vagal reduction. On the other hand, LFnu (a reflection of sympathetic influence, Pagani et al., 1997; Montano et al., 1994) increased in Dlow compared to the other groups. However, further work with recording of sympathetic activity is needed to determine whether vagal reduction is associated with an increase in sympathetic activity after SD.

It is unclear how hippocampal low levels of BDNF (reflected by low circulating BDNF) is related to autonomic disorders in Dlow and Dhigh. One possibility is that downstream structures are affected by the oxidative stress caused by the reduced levels of BDNF value (Bouvier et al., 2017). In line with this, we previously found that long-term activation of the dorsomedial nucleus of the hypothalamus (a key structure of the defense reaction) by social defeat led to the activation of 5-HT_3_ activation in the nucleus of the tracts solitarius that reduced the vagal component of the autonomic nervous system in these defeated animals (Sévoz-Couche et al., 2013). However, it should be noted that psychosocial stress induces a reduction of BDNF levels in cardiomyocytes, that seems to secondarily trigger behavioural deficits and induce hippocampal remodeling (Agrimi et al., 2019). Because all cardiovascular alterations in Dhigh recovered earlier (after day 5 for LF/HF, SBP and DBP; after day 11 for HF and after day 15 for BRS and VPBs) than circulating BDNF levels (after day 20, Brouillard et al., 2019), we can't exclude that cardiac disorder in our study may result in hippocampal BDNF depletion. Further work on cardiac and central levels of BDNF would help to answer this question.

Taking into account telemetric data, all defeated animals were vulnerable to autonomic dysfunction, but only animals with persistent lower BDNF levels (Dlow) were vulnerable to long-lasting cardiovascular alteration. In particular, the association of parasympathetic reduction associated to cardiac hypertrophy, after social defeat, is a risk factor to develop ventricular premature beats during and few days after the stress procedure (Sgoifo et al., 1998; Stilli et al., 2001). We identified ventricular ectopic events throughout the course of the study. We found that both Dlow and Dhigh started showing VPBs a few days following the social procedure (from day 5 in Dlow and day 9 in Dhigh). VPBs persisted until day 29 in Dlow but their number was higher in Dhigh than in ND until day 15 only. Therefore, we confirmed that defeated animals were vulnerable to arrhythmias, but only the recurrence of cardiac events in Dlow was associated with long lasting autonomic dysfunction. The results in Dhigh are particularly interesting. Fluctuations of VPBs in Dhigh followed perfectly the recovery in HF and BRS, more than that (shorter) of LF/HF, DBP and SBP.

Taking into account our results, we suggest that reduction of cardiac parasympathetic modulation plays an important role in maintaining sustained ventricular ectopic beats. Future studies should investigate the central mechanisms linking vulnerability to depression (Dlow, see in Blugeot et al., 2011) to cardiac parasympathetic modulation.

## 5 Conclusions

Social defeat induced long term changes in cardiac autonomic balance, with a particular importance of lowering parasympathetic efficiency, associated to increased relative LV weight, in animals that maintain lower BDNF levels. We propose that vagal withdrawal is the cause of ventricular ectopic events occurrence, and that it may be a reliable biomarker for cardiac events after stress. From a clinical point of view, these results plead for a particular attention to identify early vagal deficiency in cardiovascular diseases (La Rovere et al., 2011) or in diseases including mood disorders (Davydov et al., 2007) or cancers (Giese-Davis et al., 2015) in which autonomic dysfunction is known to be of bad prognosis.

## Funding

This work was supported by grants from INSERM and 10.13039/501100005737UPMC.

## Appendix A Supplementary data

Supplemental Fig. 1 **Dlow present a normal stress state at the end of the procedure**. A-D. Evaluation of the anxious profile in the elevated plus maze test (EPM) at day 9 and day 29. At day 9, Dlow and Dhigh animals spent more time in the closed arms (A) and less time in the open arms (B) than ND rats. At day 29, no differences were seen anymore between the three groups of rats. Time spent in center arms was similar between groups in day 9 or day 29 (C). Total number of entries (D) was lower in Dlow at day 9 only. Box and whisker graphs indicate the minimum and maximum values and the median. **p < 0.01 and ***p < 0.001 versus ND rats (Bonferroni's post hoc analysis). E. Evaluation of the hypothalamic-pituitary-adrenal axis at day 30: adrenal gland weight calculated relative to body weight was similar in ND, Dlow and Dhigh animals. Box and whisker graphs indicate the minimum and maximum values and the median. Supplemental Fig. 2. **Dlow present abnormal cardiovascular parameters and spontaneous baroreflex response in anesthetized rats at day 30**. HR (A) was similar between Dlow, Dhigh and ND. MBP (B) was higher and BRS (C) was lower in Dlow than in Dhigh or ND. Box and whisker graphs indicate the minimum and maximum values and the median. ***p < 0.01 versus ND; ^###^p < 0.01 versus Dlow. Supplemental Fig. 3. **Dlow present abnormal frequential HRV in anesthetized rats at day 30**. LF/HF ratio (A) and LFnu (D) were higher, and HF (C) and HFnu (E) were lower in Dlow than in Dhigh or ND. (B) LF tended to increase in Dlow compared to Dhigh and ND but the result was not statistically different. Box and whisker graphs indicate the minimum and maximum values and the median. *p < 0.05 and **p < 0.01 versus ND; ^#^p < 0.05 and ^###^p < 0.01 versus Dlow. Supplemental Fig. 1
