# Supplementary figures and images for: Social defeat: Vagal reduction and vulnerability to ventricular arrhythmias
Source: Neurobiol Stress. 2020 Aug 3;13:100245. doi: 10.1016/j.ynstr.2020.100245 (PMC7739042; doi:10.1016/j.ynstr.2020.100245)

Supplemental Figure 1

A

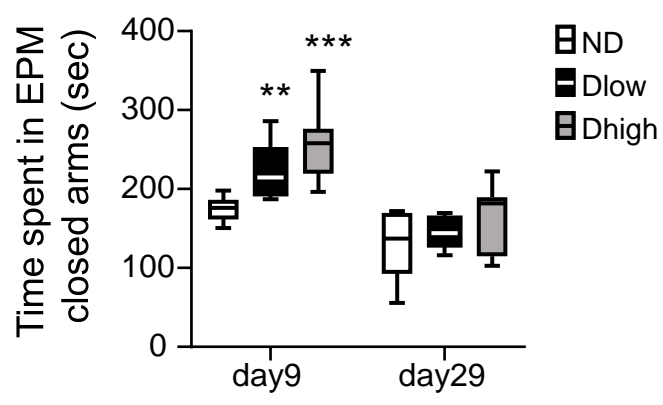

B

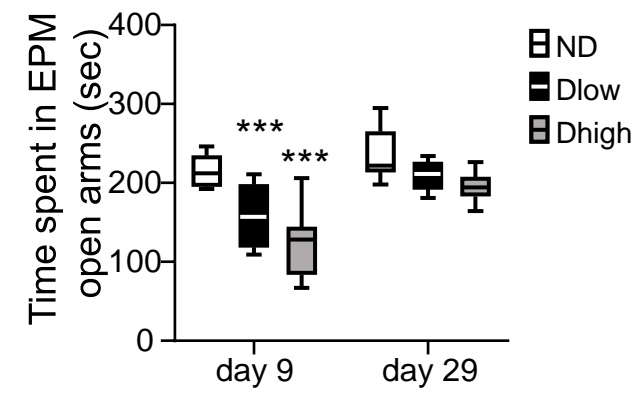

C

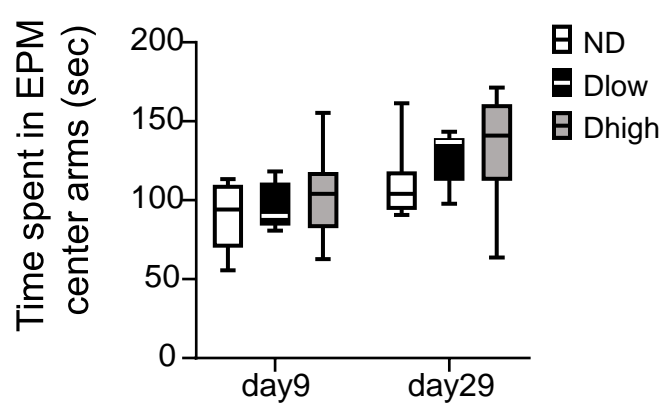

D

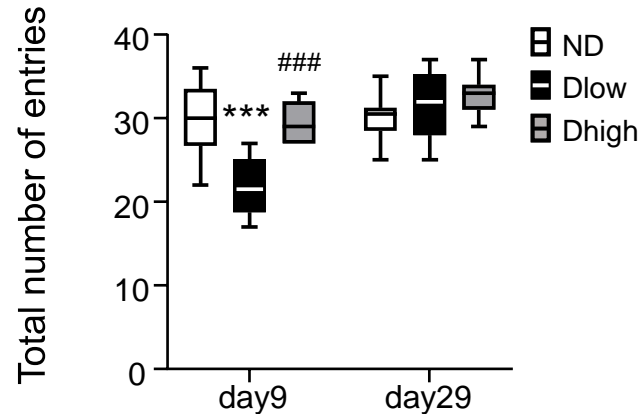

E

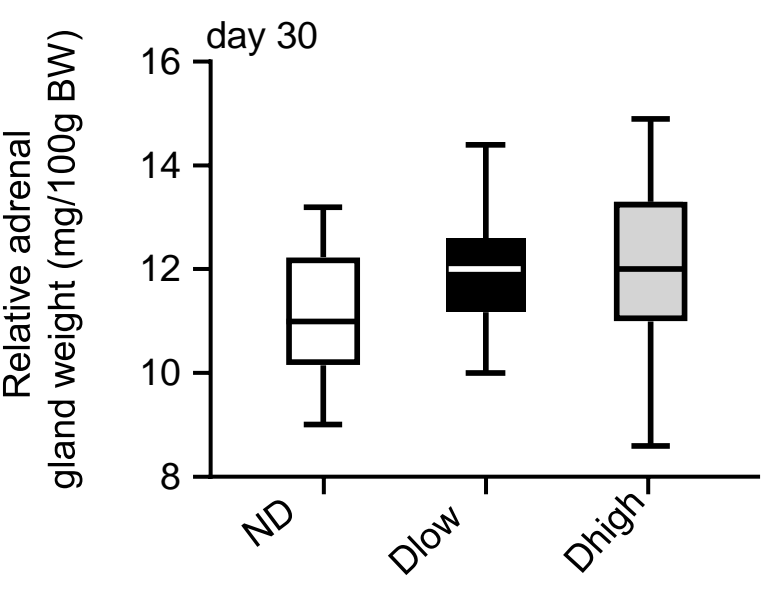

Supplemental Fig 2

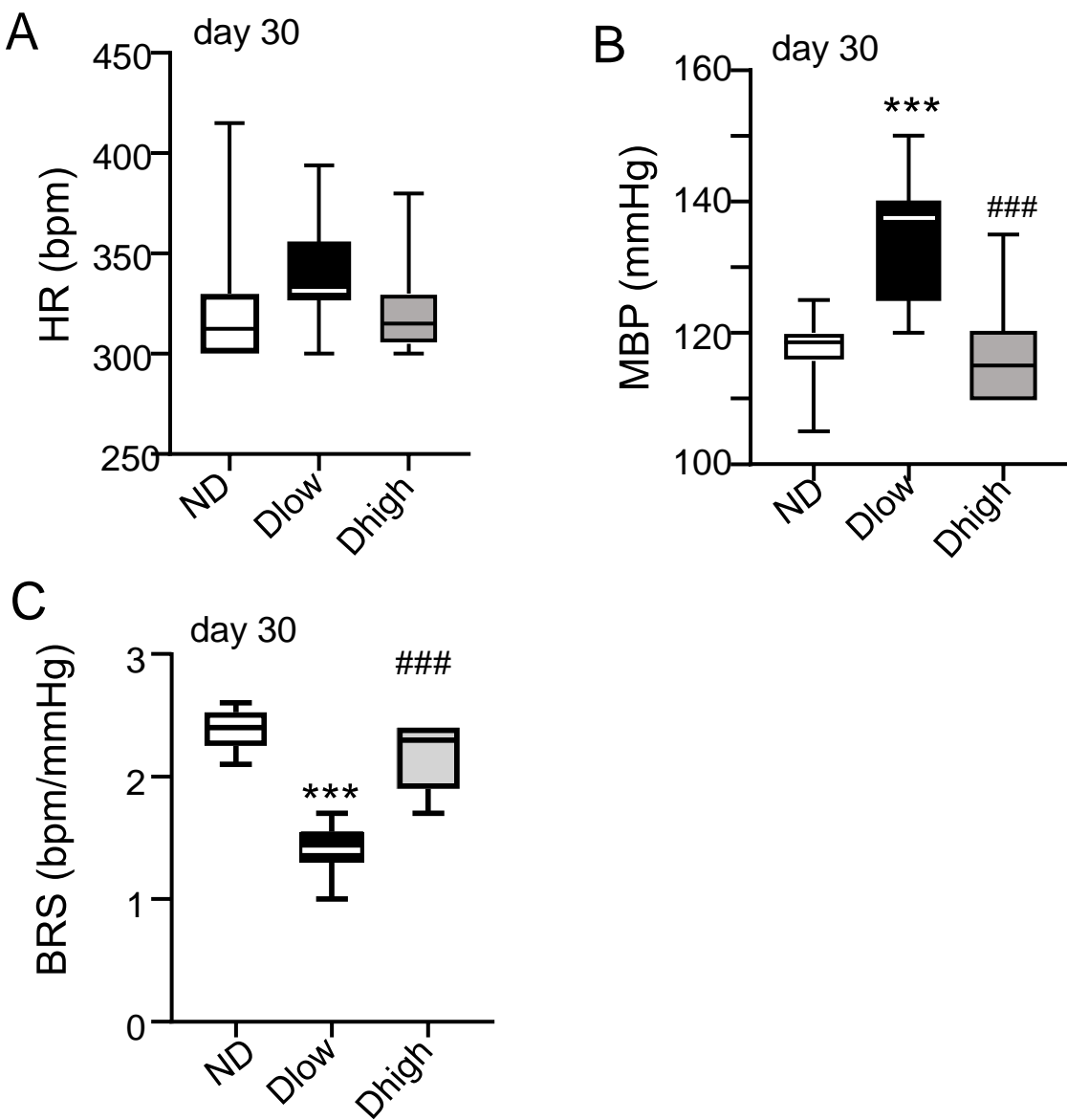

Supplemental Fig 3

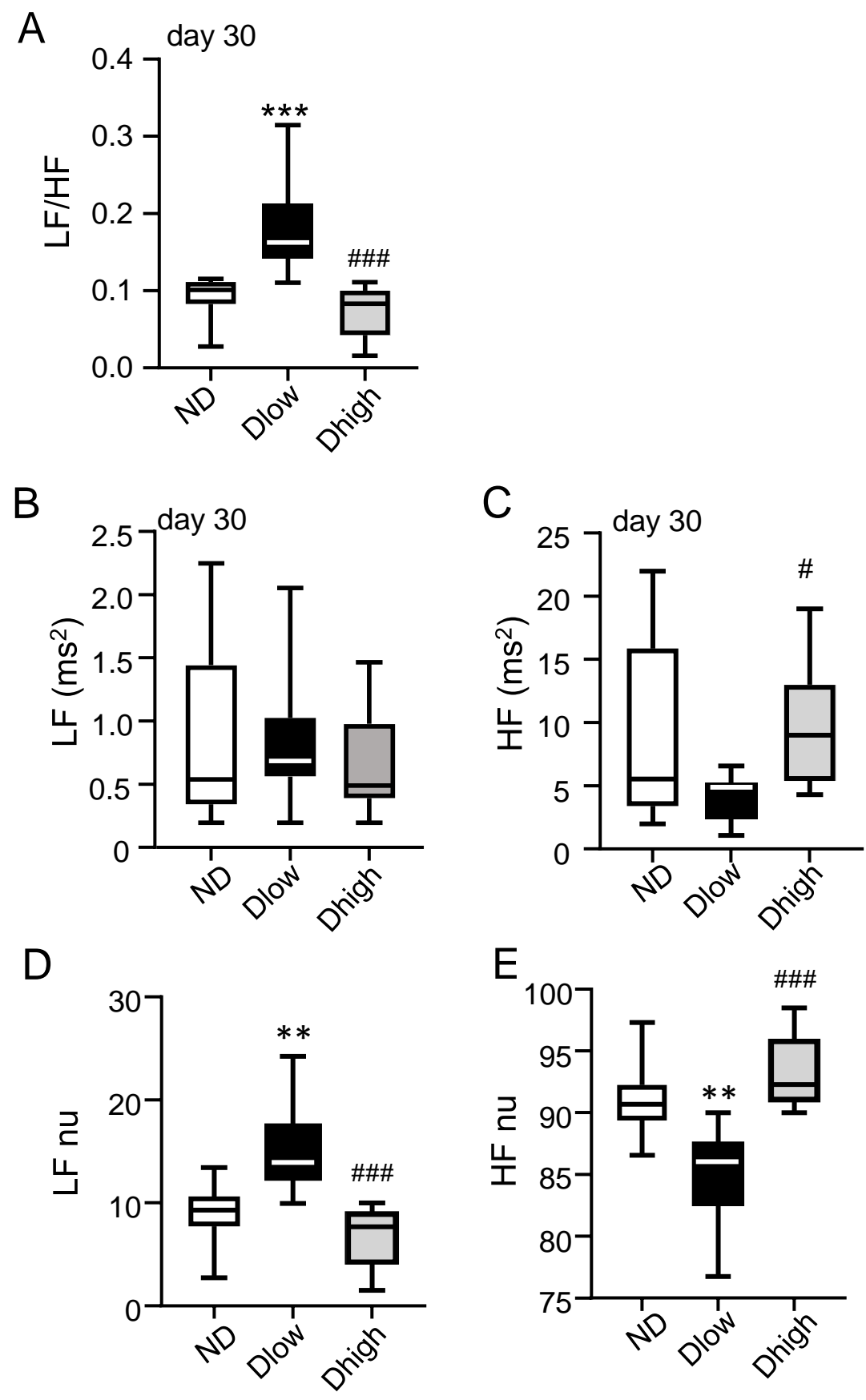

Supplement: Supplemental Fig. 1 — Dlow present a normal stress state at the end of the procedure. A-D. Evaluation of the anxious profile in the elevated plus maze test (EPM) at day 9 and day 29. At day 9, Dlow and Dhigh animals spent more time in the closed arms (A) and less time in the open arms (B) than ND rats. At day 29, no differences were seen anymore between the three groups of rats. Time spent in center arms was similar between groups in day 9 or day 29 (C). Total number of entries (D) was lower in Dlow at day 9 only. Box and whisker graphs indicate the minimum and maximum values and the median. **p < 0.01 and ***p < 0.001 versus ND rats (Bonferroni's post hoc analysis). E. Evaluation of the hypothalamic-pituitary-adrenal axis at day 30: adrenal gland weight calculated relative to body weight was similar in ND, Dlow and Dhigh animals. Box and whisker graphs indicate the minimum and maximum values and the median. Supplemental Fig. 2. Dlow present abnormal cardiovascular parameters and spontaneous baroreflex response in anesthetized rats at day 30. HR (A) was similar between Dlow, Dhigh and ND. MBP (B) was higher and BRS (C) was lower in Dlow than in Dhigh or ND. Box and whisker graphs indicate the minimum and maximum values and the median. ***p < 0.01 versus ND; ###p < 0.01 versus Dlow. Supplemental Fig. 3. Dlow present abnormal frequential HRV in anesthetized rats at day 30. LF/HF ratio (A) and LFnu (D) were higher, and HF (C) and HFnu (E) were lower in Dlow than in Dhigh or ND. (B) LF tended to increase in Dlow compared to Dhigh and ND but the result was not statistically different. Box and whisker graphs indicate the minimum and maximum values and the median. *p < 0.05 and **p < 0.01 versus ND; #p < 0.05 and ###p < 0.01 versus Dlow. [file mmc1.pdf]
